# Temperance and Longevity

**Published:** 1884-02

**Authors:** 


### Temperance and Longevity.

Mr. H. B. Robinson, at the British Association, has been urging, from the experience of life assurance offices, the value of abstinence from strong drinks as a means of prolonging human life and reducing the premiums for life insurance. He said:

"There were several mutual life assurance societies which kept quite separate the statistics of the lives of the general section and of those persons who abstained from strong drinks. At present many difficulties presented themselves in the inquiry, which would no doubt be eliminated in future years. The most valuable facts were furnished by the United Kingdom Temperance and General Provident Institution, showing that in seventeen years the claims in the temperance section were only a little over 70 per cent. of the expectancy, while in the general section they were but slightly below the expectancy. The experience of the Whittington Life Assurance Company was not yet enough to form any exact opinion upon, but the company said that 'teetotalism seems to be favorable to longevity.' The Sceptre Life Association stated that during the eighteen years of their history ended in 1882 they had 116 deaths in their temperance section against 270 expected deaths, and in 1883 the same disproportion prevailed, as they had had 51 deaths, and only seven of them were the lives of abstainers, whereas to be equal with non-abstainers there should have been 19. In the Emperor Life Assurance office, lives in the temperance branch were assured at a less rate than moderate drinkers. In some accidental offices the assumed superior lives of abstainers was recognized by a charge of 20 per cent. less to teetotalers than to moderate drinkers."

Such statements are not new, but they are very interesting. They will have to be based on a longer and larger experience before any very positive conclusions can be drawn from them; but, taken in connection with the higher premiums exacted of publicans, and their early deaths, they show temperance to great advantage. As one of the speakers in the discussion observed, it is difficult to know how the offices guarantee the abstinence of those whom they insure. There is a considerable temptation to fraud in offering a premium of 20 per cent. less to teetotalers. We do not in any way wish to imply doubt as to the advantages of the strictest temperance. We are constantly expressing our conviction that many people who regard themselves as moderate drinkers are unconsciously laying the foundation of disease. But we want strict facts on both sides, believing that there is a great amount of drinking due to ignorance, and that there are numbers of persons to be reached by intelligent and moderate statements who cannot be converted by sensational ones.—*Lancet.*

---

In praising the skill of her family physician recently, a German lady said that "he was professor of obstructions in his college."

